# Research on the Flexural Behavior of Hybrid Fiber-Reinforced BFRP Lightweight Aggregate Concrete Beams

**DOI:** 10.3390/ma19122476

**Published:** 2026-06-09

**Authors:** Biao Zhang, Jiakun Zhu, Xiaochun Fan

**Affiliations:** 1 Hubei Hongchang Architectural Decoration Engineering Co., Ltd., Wuhan 430000, China; zhangbiaopute2006@126.com; 2 The College of Post and Telecommunication, Wuhan Institute of Technology, Wuhan 430070, China; 3 School of Civil Engineering and Architecture, Wuhan University of Technology, Wuhan 430070, China; fxcfree@126.com

**Keywords:** lightweight aggregate concrete, BFRP bars, hybrid fibers, flexural behavior, crack control

## Abstract

To simultaneously address the deterioration of mechanical properties in lightweight aggregate concrete (LAC) and the insufficient deformation control capacity of hybrid fiber-reinforced polymer (BFRP) bars, an experimental study on the flexural behavior of hybrid fiber-reinforced BFRP-LAC beams was conducted. A total of eight beams with dimensions of 120 mm × 200 mm × 2000 mm were fabricated. The effects of hybrid fibers and BFRP reinforcement ratio on the flexural performance were investigated. Four-point bending tests were performed to analyze the failure modes, load–deformation responses, crack development patterns, and sectional strain distributions. The results indicate that two failure modes were experimentally observed in the BFRP-reinforced hybrid fiber LAC beams, namely concrete crushing and BFRP bar rupture, whereas balanced failure was considered a theoretical failure condition. The failure mode was strongly dependent on the reinforcement ratio. At a low reinforcement ratio (ρ = 0.68%), tensile failure governed by BFRP bar rupture occurred. At a moderate reinforcement ratio (ρ = 1.02%), a relatively ductile concrete-crushing failure was observed. When the reinforcement ratio increased to 1.56% and 1.81%, brittle concrete-crushing failure dominated. The incorporation of hybrid fibers improved the ductility and optimized the failure process. Both the hybrid fiber content and the BFRP reinforcement ratio significantly influenced the load-carrying capacity and deformation behavior of the beams. Increasing the fiber content enhanced the cracking load and ultimate load, delayed crack propagation, and reduced crack width, whereas increasing the reinforcement ratio was more effective in improving the ultimate capacity. The load–deflection curves exhibited a typical two-stage response without a yielding plateau. The bridging effect of hybrid fibers effectively mitigated stiffness degradation and improved crack control performance. Moreover, the plane section assumption was validated for hybrid fiber-reinforced BFRP-LAC beams. This study provides a technical basis for enhancing the performance of LAC and promoting the application of BFRP bars in structural engineering.

## 1. Introduction

The rapid development of urbanization and infrastructure construction has led to an increasing demand for structural materials, accompanied by higher structural self-weight and resource consumption [1]. Lightweight aggregate concrete (LAC), produced by replacing normal-weight aggregates with lightweight aggregates such as expanded clay, shale, or pumice, has been widely recognized as an effective solution to reduce structural dead load and improve structural efficiency [2,3,4,5]. In addition to weight reduction, LAC offers advantages including improved thermal insulation, enhanced seismic performance, and reduced foundation demand, which are beneficial for high-rise buildings and long-span structures [6]. However, due to the porous nature and relatively low strength of lightweight aggregates, LAC generally exhibits inferior mechanical properties compared with normal-weight concrete, including reduced compressive strength, lower elastic modulus, and increased brittleness [7,8]. Furthermore, the weak interfacial transition zone and higher water absorption of lightweight aggregates may lead to earlier cracking and larger deformation, which limit the wider structural application of LAC [9,10].

In parallel, fiber-reinforced polymer (FRP) bars, particularly basalt fiber-reinforced polymer (BFRP) bars, have attracted increasing attention as alternatives to conventional steel reinforcement in concrete structures [11]. BFRP bars offer several advantages, including high tensile strength, excellent corrosion resistance, and good durability in aggressive environments such as marine and coastal regions [12,13]. These characteristics make BFRP bars especially suitable for sustainable infrastructure applications. Nevertheless, unlike steel reinforcement, BFRP bars exhibit a linear elastic behavior up to failure and possess a relatively low elastic modulus, which often results in larger deflections and wider cracks in reinforced concrete members [14,15].

When BFRP bars are combined with LAC to form structural members, several critical issues become more pronounced. The low elastic modulus of BFRP bars results in insufficient stiffness and limited deformation control, while the relatively low strength and high porosity of LAC further reduce the stiffness and increase the brittleness of the concrete matrix. Consequently, BFRP-reinforced LAC beams tend to exhibit larger deflections, wider crack widths, and more brittle failure characteristics compared with conventional concrete beams [16,17,18,19]. To mitigate these drawbacks, the incorporation of fibers into LAC has been proven to be an effective strategy. Fiber reinforcement can enhance crack resistance, improve post-cracking behavior, and increase ductility through mechanisms such as crack bridging and stress redistribution [20,21,22]. In particular, hybrid fiber systems, which combine different types of fibers, have shown superior performance in controlling crack propagation and improving overall mechanical behavior compared to single-fiber systems [23,24,25]. For example, Liu et al. studied LAC incorporating carbon and polypropylene fibers and reported improved compressive performance and a validated analytical stress–strain model [26]. Libre et al. reported that hybrid steel and polypropylene fibers can enhance the ductility and energy absorption capacity of pumice lightweight aggregate concrete, with steel fibers playing the dominant reinforcing role [27]. Despite these advancements, existing studies on BFRP-reinforced LAC beams have predominantly focused on the use of single-type fibers, while research on hybrid fiber-reinforced systems remains relatively limited [28,29]. The synergistic effects of different fibers in improving the flexural behavior and crack control of BFRP-reinforced LAC beams have not been fully understood. Therefore, further investigation is necessary to explore the potential of hybrid fiber reinforcement in addressing the combined deficiencies of BFRP bars and LAC.

In this study, an experimental investigation is conducted to evaluate the flexural behavior of BFRP-reinforced hybrid fiber LAC beams. The effects of hybrid fiber content and BFRP reinforcement ratio on failure modes, load–deformation response, crack development, and strain distribution are systematically examined through four-point bending tests. The findings aim to provide a theoretical and experimental basis for improving the structural performance of LAC and promoting the practical application of BFRP bars in sustainable construction.

## 2. Materials and Methodology

### 2.1. Raw Materials

**Figure 1:**
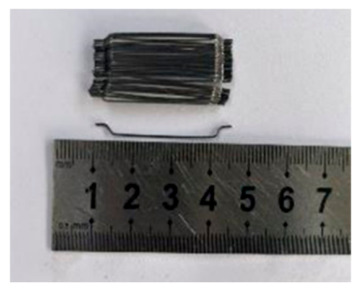
Industrial steel fiber.

**Figure 2:**
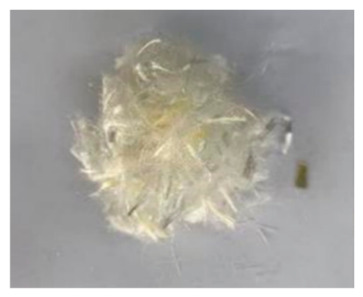
Polyvinyl alcohol (PVA) fiber.

**Figure 3:**
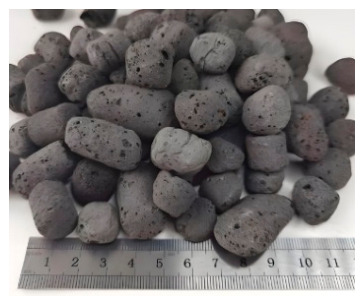
Expanded shale lightweight aggregate.

Ordinary Portland cement (P.O 42.5) supplied by Hubei Huaxin Cement Company was used as the cementitious material. End-hooked industrial steel fibers with a length of 35 mm, provided by Hebei Demai Wire Mesh Products Company, were employed to enhance mechanical performance (see Figure 1). Additionally, high-strength polyvinyl alcohol (PVA) fibers manufactured by Shanghai Chenqi Chemical Technology Co., Ltd., (Shanghai, China), were incorporated, as illustrated in Figure 2. The lightweight aggregate used in this study was 800-grade crushed expanded shale ceramsite supplied by Yichang Guangda Co., Ltd., (Yichang, China), as shown in Figure 3. The aggregates were produced from shale rich in silica and alumina through crushing, pelletizing, preheating, and high-temperature sintering. The raw shale was crushed to particles smaller than 10 mm and pelletized into spherical or ellipsoidal particles with diameters of 5–20 mm. The pellets were preheated at 200–400 °C and subsequently sintered at 1100–1300 °C, during which internal gases expanded to form a porous structure. After sintering, the aggregates were rapidly cooled to stabilize the pore system. The expanded shale ceramsite features low density, relatively high strength, and good sustainability. The basic physical properties are summarized in Table 1.

Natural river sand was used as the fine aggregate. The longitudinal reinforcement and stirrups of the beam specimens were fabricated using HPB300 plain round steel bars with a diameter of 8 mm. Basalt fiber-reinforced polymer (BFRP) bars, supplied by Jiangsu Green Material Valley New Material Technology Development Co., Ltd., (Nanjing, China), were used as the main reinforcement, with nominal diameters of 8, 12, 14, and 16 mm. The rib height of the BFRP bars was approximately 6% of their diameter, and the length was 1980 mm. The mechanical properties of the BFRP bars are summarized in Table 2. A polycarboxylate-based high-performance superplasticizer supplied by Hunan Zhongyan Building Materials Technology Co., Ltd., (Changsha, China), was used in this study, with a solid content of 40% and a water-reducing efficiency exceeding 25%.

### 2.2. Experimental Methods

Previous studies and preliminary mix trials have demonstrated that a hybrid combination of steel fibers and PVA fibers with a volume ratio of 1:1 yields optimal basic mechanical performance for LAC [30,31]. The suitable total hybrid fiber content is generally in the range of 0.6–1.5% by volume. When the fiber content exceeds 1.5%, a deterioration in mechanical properties is observed due to fiber agglomeration and reduced workability. Based on these findings, the present study adopts a hybrid fiber reinforcement strategy for LAC, in which steel fibers and PVA fibers are incorporated at a fixed volume ratio of 1:1, with the total fiber content varying between 0.6% and 1.5%. The experiment was designed to investigate the effects of hybrid fiber (HF) content and BFRP reinforcement ratio. A total of eight beam specimens with dimensions of 120 mm × 200 mm × 2000 mm were fabricated, including one control specimen without fiber reinforcement for comparison. The concrete cover was set to 15 mm in accordance with Class I environmental requirements. Stirrups were not provided in the pure bending section, whereas 8 mm diameter stirrups were arranged at a spacing of 100 mm outside the pure bending region. The mix design was developed based on commonly adopted proportions reported in previous studies on LAC [5,6,7,8,9]. The detailed mix proportion of the LAC without fibers are presented in Table 3, including the amounts of cement, water, fine and coarse aggregates, and chemical admixture. The 28-day cubic compressive strength of the concrete was measured as 53 MPa.

**Figure 4:**
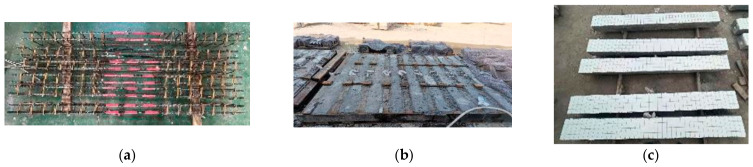
Fabrication process of beam specimens: (**a**) reinforcement assembly and strain gauge installation on BFRP bars; (**b**) specimen casting and curing; (**c**) grid marking on specimen surface.

The beam specimens were fabricated using timber formwork, with multiple molds fixed side-by-side to ensure dimensional accuracy and prevent deformation. Reinforcement cages were assembled by tying stirrups and longitudinal bars according to the design requirements. Strain gauges (BX120-3AA) were installed on the longitudinal reinforcement at the midspan and at two points 150 mm away from the midspan, resulting in six measurement points per beam. Prior to installation, the bar surface was ground, polished, cleaned with alcohol, and then bonded with strain gauges using adhesive, followed by protective wrapping with gauze and electrical tape. Concrete was cast after placing spacers to ensure the required cover thickness, and the specimens were vibrated to eliminate entrapped air. After 24 h, the molds were removed and the specimens were cured under outdoor conditions at an average temperature of 20–25 °C. During the curing period, the specimens were covered with wet burlap and periodically sprayed with water to maintain sufficient moisture. Finally, the surface was whitened using a gypsum-based coating, and a 50 mm grid was marked for crack observation. The overall fabrication process is shown in Figure 4.

The basic parameters of the beam specimens are summarized in Table 4. In the designation, “LAC” represents lightweight aggregate concrete, and the following number indicates the total content of hybrid fibers; “B” indicates that the lower longitudinal reinforcement is BFRP, followed by a number representing its diameter; “Φ” denotes HPB300 steel reinforcement. For example, “LAC6-B12” denotes a beam specimen with a hybrid fiber content of 0.6% and two 12 mm BFRP bars provided as bottom longitudinal reinforcement.

**Figure 5:**
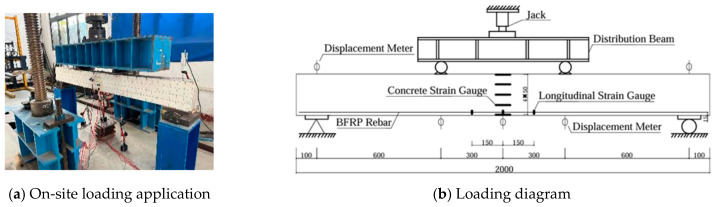
Schematic of the testing device.

A four-point bending test was conducted, with the hydraulic loading device applied at the mid-span. The load was evenly transmitted to the beams over 1/3 of the clear span through a distribution beam. Three high-range displacement transducers were installed at the mid-span and at positions 300 mm on either side of the mid-span. Small-range displacement transducers were placed on top of the supports at both ends to record the mid-span deflection and support displacements, respectively. Strain gauges were embedded in the lower longitudinal reinforcement during fabrication to measure its strain. A schematic of the testing setup is shown in Figure 5.

A preloading procedure was applied to ensure proper contact between the specimens and the loading system. A maximum preload of 2 kN was applied in 1 kN increments at a rate of 5 kN/min, with each step held for 3 min before unloading. Any surface gaps were leveled using fine sand if necessary. The formal loading test was conducted under load-controlled conditions. Prior to cracking, loading was applied in 2 kN increments at 5 kN/min with a 3 min holding period. After cracking, the load increment was increased to 5 kN with a 5 min holding time to facilitate crack observation and measurement. Failure was defined by one or more of the following criteria: rapid increase in displacement, load stagnation, rupture of tensile reinforcement, crack width exceeding 1.5 mm, deflection reaching L/50, or crushing of the compression zone. The measured parameters included load, deflection, concrete strain, reinforcement strain, and crack development. Load was recorded by a 100-t testing machine, while deflection was measured using LVDTs. Strain data were collected using embedded and surface-mounted strain gauges connected to a TST3822EW acquisition system with temperature compensation. Crack development was monitored using a grid method, and crack widths were periodically measured using a crack width microscope.

## 3. Results and Discussion

### 3.1. Failure Mode

Based on the observed failure sequences and characteristics of the BFRP bars and concrete, the eight test beams exhibited three typical failure modes:

(1) BFRP rebar tensile failure (BTF)

**Figure 6:**
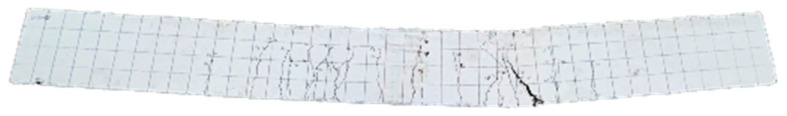
Failure mode of specimen LAC12-B8.

This failure mode was observed in specimen LAC12-B8, with a low reinforcement ratio and hybrid fiber content, as shown in Figure 6. In the initial loading stage, flexural cracks first developed in the midspan region and gradually propagated upward. As loading increased, additional flexural cracks formed in the pure bending zone, and one of the early cracks evolved into the dominant crack. Subsequently, diagonal cracks appeared in the shear span and progressively extended toward the compression zone, while crack width and crack density increased with loading. Before failure, crack development became more pronounced, and deformation localized in the midspan region. The specimen ultimately failed due to sudden rupture of the tensile BFRP reinforcement, accompanied by an audible fracture sound. At failure, the concrete in the compression zone remained largely intact, indicating that crushing of concrete did not govern the failure process. This failure mode is characterized as a tension-controlled, under-reinforced behavior. It is primarily governed by the tensile capacity of the BFRP reinforcement, exhibiting relatively higher deformation capacity compared with compression-controlled failure modes.

(2) Crushing brittle failure of concrete in the compression zone (CBF)

**Figure 7:**
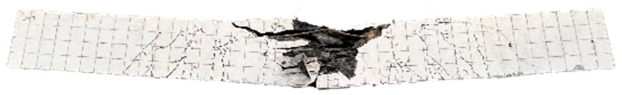
Failure mode of specimen LAC0-B12.

**Figure 8:**
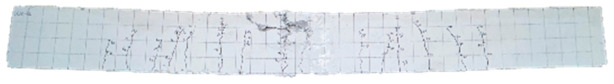
Failure mode of specimen LAC12-B14.

**Figure 9:**
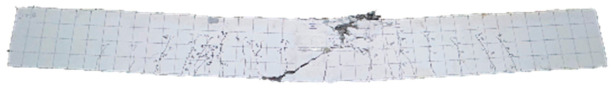
Failure mode of specimen LAC12-B16.

This failure mode was observed in specimens LAC0-B12, LAC12-B14, and LAC12-B16, as shown in Figure 7, Figure 8 and Figure 9. It is characterized by a compression-controlled behavior with relatively stable flexural crack development during the loading process. At the initial stage, vertical flexural cracks formed in the pure bending region and gradually propagated upward, while multiple diagonal cracks developed in the shear span as loading increased. Crack density became progressively higher, and crack widths enlarged with increasing load. After a certain stage, crack propagation tended to stabilize, with no significant formation of new cracks, while existing cracks continued to widen and extend. As loading approached the ultimate state, severe crushing and spalling of concrete occurred in the compression zone near the loading area and at the midspan region. The concrete cover layer was progressively damaged and locally detached, accompanied by bulging and loss of integrity in the compression zone. The failure was ultimately governed by crushing of the concrete in the compression zone rather than rupture of the BFRP reinforcement, which remained intact at failure. This indicates a typical over-reinforced or compression-controlled failure mode, characterized by limited ductility and sudden loss of load-carrying capacity once the compressive concrete reached its ultimate strain.

(3) Crushing ductile failure of concrete in the compression zone (CDF)

**Figure 10:**
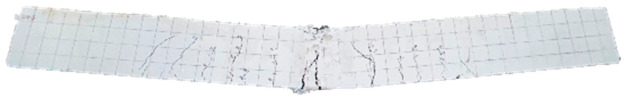
Failure mode of specimen LAC6-B12.

**Figure 11:**
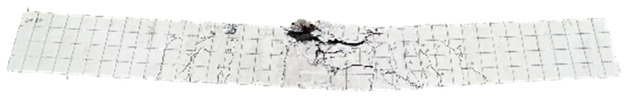
Failure mode of specimen LAC9-B12.

**Figure 12:**
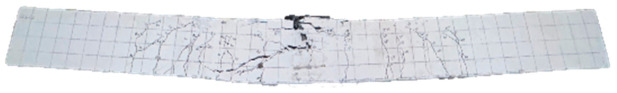
Failure mode of specimen LAC12-B12.

**Figure 13:**
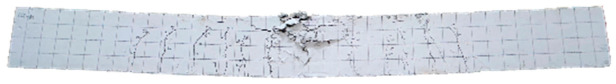
Failure mode of specimen LAC15-B12.

This failure mode was observed in specimens LAC6-B12, LAC9-B12, LAC12-B12, and LAC15-B12, as shown in Figure 10, Figure 11, Figure 12 and Figure 13. It is characterized by progressive flexural cracking followed by compression-controlled crushing at ultimate state, while maintaining relatively stable deformation development during the loading process. In the early loading stage, flexural cracks initiated in the midspan region and gradually propagated upward. With increasing load, multiple vertical cracks developed in the pure bending zone, while diagonal cracks appeared and propagated in the shear span. Crack number and crack width increased progressively, and one or more cracks eventually became dominant. In the intermediate stage, crack propagation accelerated and crack distribution became denser, accompanied by continuous stiffness degradation and increasing midspan deflection. As loading approached failure, crack widths further increased and deformation became more pronounced, while crack propagation gradually stabilized. Eventually, crushing and spalling of concrete occurred in the compression zone near the loading area and midspan region. The failure was governed by compressive crushing of concrete rather than rupture of BFRP reinforcement, which remained unbroken at failure. Compared with pure compression-controlled brittle failure, this failure mode exhibited relatively better deformation capacity and energy dissipation ability due to the presence of hybrid fibers, indicating an improved but still compression-governed failure mechanism.

Based on the experimental observations, two failure modes were experimentally observed in the BFRP-reinforced hybrid fiber LAC beams, namely concrete crushing and BFRP bar rupture. Due to the linear-elastic behavior of BFRP bars without yielding, both tensile and compression failure modes are brittle in nature, while balanced failure represents a theoretical transitional state between the two limit conditions. In this study, only tensile rupture and compression crushing failure modes were observed experimentally. No balanced failure was captured within the tested range of reinforcement ratios and fiber contents. Tensile failure is characterized by sudden rupture of BFRP reinforcement accompanied by extensive flexural cracking in the pure bending and shear regions, occurring without obvious warning signs. Compression failure is governed by crushing and spalling of concrete in the compression zone, while the BFRP bars remain intact. This mode is associated with progressive crack development and relatively improved deformation capacity due to the bridging effect of hybrid fibers. The experimental results summarized in Table 5 indicate that the failure mode is strongly dependent on the reinforcement ratio. At low reinforcement ratios, tensile rupture governs failure, while at intermediate and high reinforcement ratios, compression-controlled crushing becomes dominant. Within the present test program, no balanced failure mode was observed.

The flexural response of BFRP-reinforced hybrid fiber lightweight aggregate concrete beams is governed by the coupled action of fiber reinforcement, BFRP reinforcement, and the concrete matrix, which together determine the cracking behavior, stiffness degradation, and load-carrying mechanism. Before cracking, the concrete matrix and BFRP reinforcement jointly resist the applied tensile stress, resulting in an approximately linear load–deflection response. In this stage, the contribution of hybrid fibers is limited, and the global behavior is primarily governed by the elastic properties of concrete and BFRP bars. After cracking, the tensile resistance of concrete is gradually lost, and the load is redistributed to hybrid fibers and BFRP reinforcement. The hybrid fibers play a crucial role in bridging microcracks, transferring tensile stresses across crack interfaces, and restraining crack opening. This crack-bridging effect delays crack localization and promotes the development of multiple fine cracks rather than a few dominant cracks, thereby improving cracking resistance and post-cracking stiffness. Meanwhile, due to the relatively low elastic modulus of BFRP reinforcement, larger tensile strains are induced in the reinforcement after cracking compared with conventional steel-reinforced concrete members. This leads to a more deformation-sensitive response, where stiffness degradation is closely related to crack development and bond performance. The bond behavior between BFRP bars and lightweight aggregate concrete also significantly influences the structural response. As loading increases, interfacial micro-slip may occur between the reinforcement and surrounding concrete, especially in specimens with higher reinforcement ratios. This slip affects stress transfer efficiency and contributes to non-uniform strain distribution along the reinforcement, which is consistent with the observed variations in crack spacing and crack propagation patterns.

At the ultimate stage, the failure mode is governed by either rupture of BFRP reinforcement or crushing of concrete in the compression zone, depending on the reinforcement ratio. The transition between these two modes reflects the redistribution of internal forces and the competition between tensile capacity of BFRP bars and compressive capacity of concrete. The presence of hybrid fibers enhances energy dissipation capacity and improves damage tolerance, particularly in compression-controlled failure modes, by maintaining partial integrity of the cracked concrete matrix.

### 3.2. Load–Deformation Performance

#### 3.2.1. Characteristics of Load–Deflection Curve

**Figure 14:**
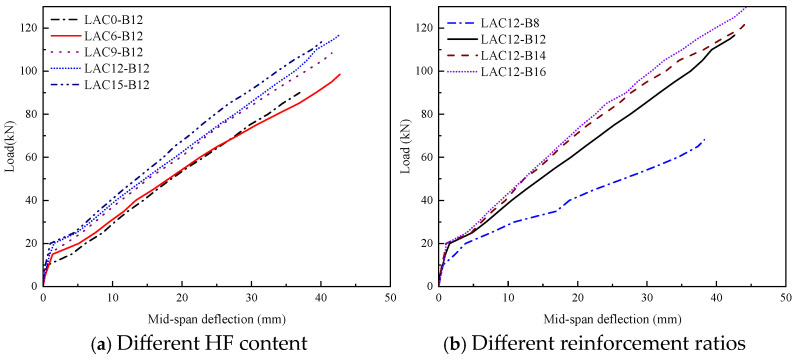
Load–midspan deflection response of the beams.

The load–midspan deflection curves of all specimens are presented in Figure 14. As shown in Figure 14a,b, the BFRP-reinforced hybrid fiber LAC beams exhibit a typical two-stage response, including an initial linear elastic stage before cracking and a post-cracking stage without a distinct yield plateau. In the initial stage, concrete and BFRP reinforcement work together to resist bending, resulting in a linear load–deflection relationship with relatively high stiffness. After cracking, the tensile resistance of concrete is lost and the BFRP bars become the primary load-bearing component, leading to a reduction in stiffness at the cracking point. However, due to the linear-elastic behavior of BFRP reinforcement, the load–deflection curves remain approximately linear in the post-cracking stage until failure.

As shown in Figure 14a, which illustrates the effect of fiber content, increasing the hybrid fiber volume fraction improves the initial cracking resistance and reduces post-cracking stiffness degradation. The beneficial effect is attributed to the crack-bridging capacity of fibers, which delays crack propagation and improves stress redistribution. However, the improvement becomes less pronounced at higher reinforcement ratios. Figure 14b presents the influence of reinforcement ratio. It can be observed that increasing the reinforcement ratio leads to a reduction in midspan deflection under the same load level, while the overall shape of the load–deflection curves remains similar. In addition, specimens governed by concrete crushing failure exhibit better deformation capacity compared to those governed by BFRP rupture failure.

#### 3.2.2. Initial Cracking and Ultimate Load of Beams

**Figure 15:**
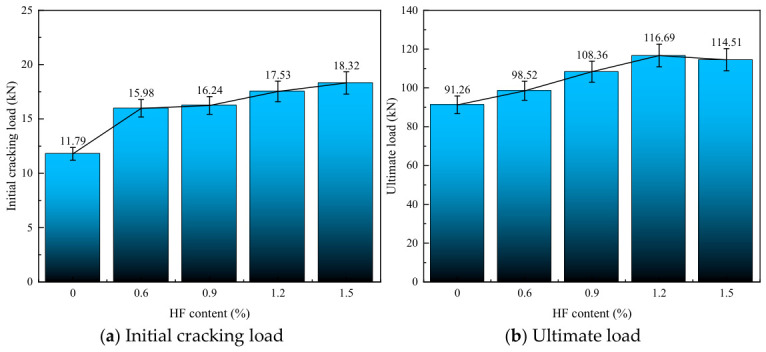
Comparison of beams with varying HF content.

**Figure 16:**
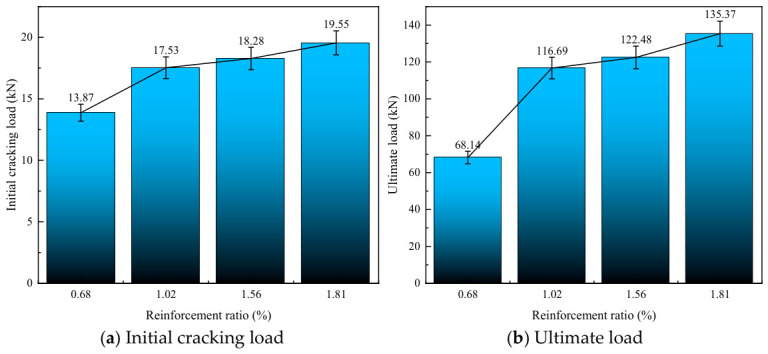
Comparison of beams with varying BFRP reinforcement ratios.

The comparison of cracking load and ultimate load under different reinforcement ratios and hybrid fiber volume fractions is shown in Figure 15 and Figure 16.

As shown in Figure 15, when the reinforcement ratio of BFRP bars is kept constant at 1.02%, the cracking load increases with the increase in HF content. The ultimate load exhibits an initial increasing trend followed by a decrease trend at higher fiber content. For the reference specimen LAC0-B12, the cracking load and ultimate load are 11.79 kN and 91.26 kN, respectively. When the HF volume fraction is 0.6%, 0.9%, 1.2%, and 1.5%, the cracking load increases by 35.54%, 37.74%, 48.69%, and 55.39%, respectively, while the ultimate load increases by 7.96%, 18.74%, 27.87%, and 25.48%, respectively. These results indicate that the incorporation of hybrid fibers improves both the cracking resistance and load-carrying capacity of lightweight aggregate concrete beams.

As shown in Figure 16, under the same HF content, both cracking load and ultimate load increase with the reinforcement ratio. For the specimen with a reinforcement ratio of 0.68%, the cracking load and ultimate load are 13.87 kN and 68.14 kN, respectively. When the reinforcement ratio increases to 1.02%, 1.56%, and 1.81%, the cracking load increases by 26.38%, 31.79%, and 40.95%, respectively, while the ultimate load increases by 71.25%, 79.75%, and 98.66%, respectively. These results indicate that increasing the reinforcement ratio trends to be more effective in enhancing the ultimate load capacity compared to cracking resistance.

### 3.3. Crack Development Characteristics

#### 3.3.1. Fracture Morphology and Distribution

**Figure 17:**
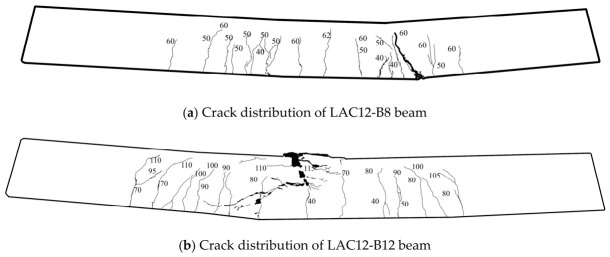
Crack distribution patterns of representative beams.

During the tests, all specimens exhibited similar crack development patterns, and a typical crack evolution process is illustrated in Figure 17. In the initial stage, vertical flexural cracks first appeared in the tensile zone of the pure bending region and gradually propagated toward the compression zone. With increasing load, additional vertical cracks continuously formed in the pure bending region, accompanied by crack growth and widening. As deflection further increased, diagonal cracks developed in the shear span, extending from the supports toward the loading points. Ultimately, all cracks propagated continuously until failure of the specimens.

Compared with the specimens without fiber reinforcement, the fiber-reinforced beams exhibited distinct differences in crack development behavior. In the non-fiber specimens, the initial cracks rapidly propagated to approximately three-quarters of the beam depth, with faster crack growth and more pronounced crack extension. Under the same load level, the fiber-reinforced specimens showed fewer cracks with smaller spacing and more limited crack propagation, indicating that fibers effectively restrained crack initiation and development through a crack-bridging mechanism. Furthermore, with increasing reinforcement ratio, the specimens exhibited a higher number of cracks with reduced spacing, leading to a more uniformly distributed crack pattern. It is also noted that in specimens with higher reinforcement ratios, cracks tended to propagate horizontally during the later stage of loading, which can be attributed to interfacial slip between the BFRP reinforcement and concrete.

#### 3.3.2. Crack Width Control

**Figure 18:**
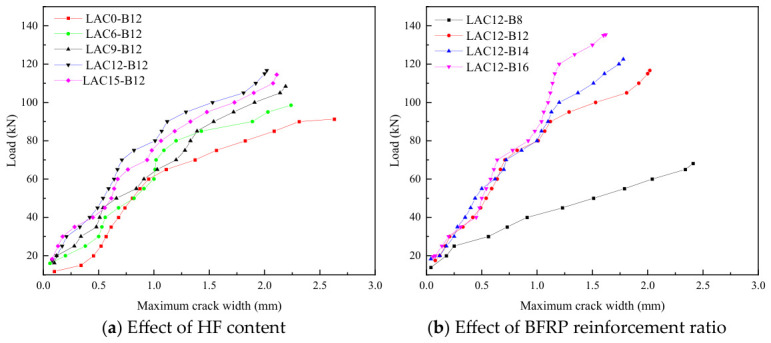
Effect of HF content and BFRP reinforcement ratio on maximum crack width of beams.

The relationship between maximum crack width and applied load for all specimens is shown in Figure 18. It can be observed that the crack width of all specimens increases progressively with increasing load.

Figure 18a presents the load–maximum crack width relationships under different hybrid fiber volume fractions. The results indicate that fiber-reinforced specimens exhibit significantly reduced crack widths compared with the plain concrete specimen. Under the same load level, the maximum crack widths of fiber-reinforced beams are reduced by 34.25%, 27.12%, 44.66%, and 41.75%, respectively. This indicates that hybrid fibers are effective in controlling crack development. The crack-bridging effect of fibers contributes to stress transfer across cracks, reducing the tensile stress carried by BFRP bars, enhancing the integrity of the cracked concrete section, and improving sectional stiffness. In addition, fibers improve the bond behavior between BFRP bars and the surrounding matrix, reducing slip and promoting stress redistribution, which leads to the formation of multiple secondary cracks and delays the propagation of the dominant crack. However, with further increase in load, fiber pull-out and interfacial degradation occur, resulting in a reduction in crack control efficiency and an accelerated crack widening rate at later stages.

Figure 18b shows the influence of reinforcement ratio on the load–maximum crack width response. The results indicate a clear inverse relationship between reinforcement ratio and maximum crack width under the same load level. When the reinforcement ratio increases from 0.68% to 1.02%, 1.56%, and 1.81%, the maximum crack width decreases by 16.18%, 26.14%, and 32.78%, respectively.

It should be noted due to the limited number of specimens, each test configuration was represented by a single specimen. Therefore, statistical evaluation of the experimental results was not possible, and the observed differences in load capacity and crack width should be interpreted as indicative trends rather than statistically validated conclusions.

### 3.4. Strain Analysis and Verification of the Plane Section Assumption

#### 3.4.1. Strain Development of BFRP Tendons

The strain values of the BFRP reinforcement measured at the midspan and at locations 150 mm from both sides of the midspan showed minor differences, with a coefficient of variation less than 5%. Therefore, the average strain of the two bottom BFRP bars was adopted as the representative experimental value.

**Figure 19:**
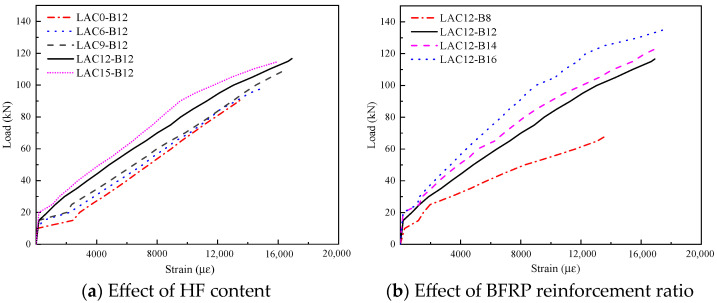
Load–BFRP tendon strain relationship for different concrete mixtures and BFRP reinforcement ratios.

As shown in Figure 19, the load–strain curves of the BFRP reinforcement at the midspan exhibit a distinct two-stage response, which is consistent with the load–deflection behavior. In the pre-cracking stage, concrete participates in tension resistance, and the BFRP strain increases approximately linearly with load at a relatively moderate rate. After cracking, the concrete in the tension zone ceases to carry tensile stress, resulting in a faster increase in BFRP strain while maintaining an approximately linear relationship with a reduced slope.

Figure 19a,b present the influence of hybrid fiber content and reinforcement ratio on the load–strain response, respectively. The results indicate that, at the same load level, the strain in BFRP bars decreases with increasing hybrid fiber content due to the stress-sharing effect of fibers, which reduces the demand on BFRP reinforcement. When the hybrid fiber content is constant, the influence of reinforcement ratio becomes more significant in the post-cracking stage. Before cracking, the effect of reinforcement ratio on BFRP strain is limited; however, after cracking, beams with higher reinforcement ratios exhibit lower strain levels and a steeper load–strain response.

Under a load level of 60 kN, the measured strains in the 8 mm BFRP bars reach 11,626 με, whereas those in the 12 mm, 14 mm, and 16 mm bars decrease to 6407 με, 5134 με, and 4385 με, respectively. This reduction is attributed to the increase in flexural stiffness with higher reinforcement ratios, which effectively limits structural deformation. Based on the measured strain data and the elastic modulus of BFRP bars, the corresponding stresses near failure are approximately 602 MPa, 624 MPa, and 677 MPa for the 12 mm, 14 mm, and 16 mm specimens, respectively, which account for only about 55–65% of their ultimate tensile strength, indicating that the tensile capacity of the BFRP reinforcement is not fully utilized.

#### 3.4.2. Strain Distribution of Concrete Section and Verification of the Plane Section Assumption

**Figure 20:**
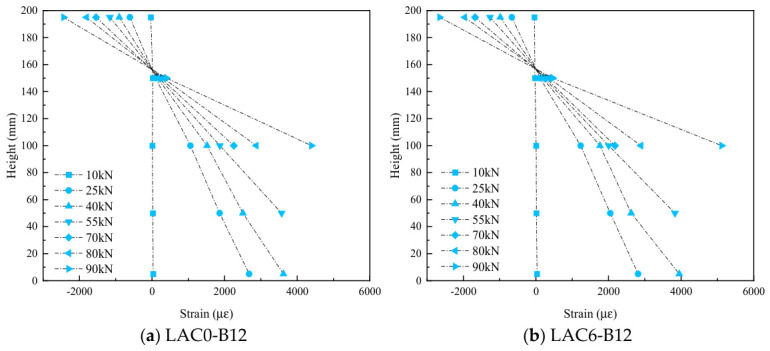

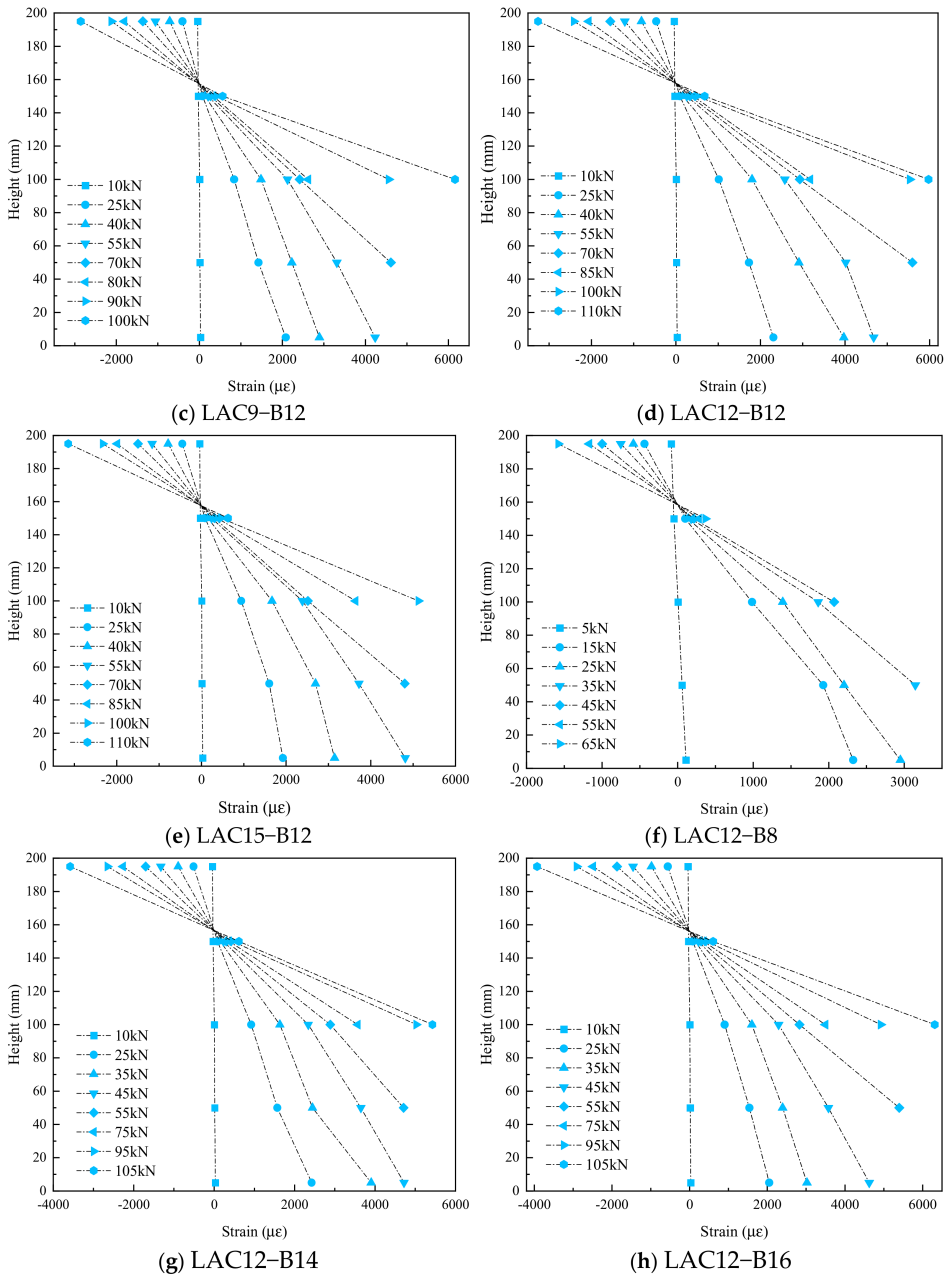
Concrete strain distribution along the mid-span section of the beams.

The concrete strain distribution in the pure bending region of all specimens is shown in Figure 20. Due to the development of cracks, some strain gauges became detached and invalid during loading; therefore, only the valid measurement points are presented. It can be observed that all specimens exhibit similar strain development patterns. The concrete in the upper region is under compression, while the lower region is under tension, and both compressive and tensile strains increase with increasing load.

The concrete strain shows an approximately linear distribution along the beam depth from the initial loading stage to failure for all specimens, indicating that the plane section assumption remains valid throughout the loading process. For the specimen with a reinforcement ratio of 0.68%, the depth of the compression zone is approximately 26.5 mm. With increasing reinforcement ratio, the compression zone depth increases to approximately 29 mm, 34 mm, and 37.5 mm, respectively, indicating that the neutral axis gradually shifts downward with increasing reinforcement ratio.

### 3.5. Theoretical and Experimental Comparison of Flexural Capacity

According to the calculation method specified in Clause 10.1.2 of the Chinese code GB 50608-2020, the flexural capacity of BFRP-reinforced concrete members at the ultimate limit state can be calculated using Equations (1)–(5) as follows:

(1) $$\xi_{\mathrm{fb}}=\frac{\beta_{1}\varepsilon_{cu}}{\varepsilon_{cu}+\frac{f_{\mathrm{fd}}}{E_{f}}}$$

(2) $$\rho_{\mathrm{fb}}=\frac{\alpha_{1}f_{c}}{f_{\mathrm{fd}}}\xi_{\mathrm{fb}}$$

(3) $$f_{\mathrm{fe}}=\left\{\begin{array}{ll} f_{\mathrm{fd}} & \;\rho_{f}<1.5\rho_{\mathrm{fb}}\\ {\left(\frac{\rho_{f}}{\rho_{\mathrm{fb}}}\right)}^{-0.55}f_{\mathrm{fd}} & \;\rho_{f}\geq 1.5\rho_{\mathrm{fb}} \end{array}\right.$$

(4) $$M\leq A_{f}\;f_{\mathrm{fe}}\left(h_{0f}-\frac{x}{2}\right)$$

(5) $$x=\left\{\begin{array}{ll} \left[\frac{0.14}{1+400\frac{f_{\mathrm{fe}}}{E_{f}}}+\frac{\rho_{f}f_{\mathrm{fe}}}{f_{c}}\right]h_{0f} & \;\rho_{f}<1.5\rho_{\mathrm{fb}}\\ \frac{\rho_{f}f_{\mathrm{fe}}}{\alpha_{1}f_{c}}h_{0f} & \;\rho_{f}\geq 1.5\rho_{\mathrm{fb}} \end{array}\right.$$

In the above equations, $\xi_{\mathrm{fb}}$ is the relative depth of the balanced compression zone; $\rho_{\mathrm{fb}}$ is the balanced reinforcement ratio; $f_{\mathrm{fd}}$ is the design tensile strength of the BFRP bars; *M* is the design bending moment (kN·m); *x* is the depth of the equivalent rectangular stress block in the concrete compression zone (mm); $f_{\mathrm{fe}}$ is the effective stress in the BFRP bars (N/mm^2^); and $\rho_{f}$ is the actual reinforcement ratio of the member.

Table 6 compares the experimentally measured flexural capacities with the theoretical predictions. The results indicate that the values calculated according to GB 50608–2020 are in good agreement with the experimental results.

## 4. Conclusions

This study experimentally investigated the flexural behavior of eight BFRP-reinforced hybrid fiber lightweight aggregate concrete beams under four-point bending, focusing on the effects of hybrid fiber content (0–1.5%) and BFRP reinforcement ratio (0.68–1.81%) on failure modes, characteristic loads, deflection, strain responses, and crack development. The main conclusions are summarized as follows:

(1) Two failure modes were experimentally observed in the BFRP-reinforced hybrid fiber LAC beams, namely concrete crushing and BFRP bar rupture, whereas balanced failure was considered as a theoretical failure condition. With a low reinforcement ratio (0.68%), tensile rupture of BFRP bars governed failure. At a moderate reinforcement ratio (1.02%), a relatively ductile compression crushing failure was observed, while at higher reinforcement ratios (1.56% and 1.81%), brittle compression crushing failure dominated. The incorporation of hybrid fibers enhanced matrix integrity, improved interfacial bonding, and increased sectional stiffness, promoting a transition from brittle to relatively ductile compression failure, although the influence became less pronounced at higher fiber contents.
(2) Both hybrid fiber addition and reinforcement ratio increase effectively improved cracking load and ultimate load, while also enhancing crack control performance. Hybrid fibers were more effective in increasing cracking resistance, whereas reinforcement ratio had a more pronounced effect on ultimate load capacity. The improvement mechanisms are mainly attributed to the crack-bridging effect of fibers and the increased flexural stiffness provided by higher reinforcement ratios.
(3) All beams exhibited a typical two-stage load–deflection response, consisting of pre-cracking elastic behavior and post-cracking behavior without a yield plateau. The addition of hybrid fibers reduced midspan deflection by improving crack resistance and delaying crack propagation. A higher reinforcement ratio resulted in lower deflection under the same load level, indicating increased structural stiffness.
(4) The load–strain response of BFRP reinforcement followed a similar two-stage pattern to the load–deflection behavior. Hybrid fiber incorporation slightly reduced BFRP strain, while reinforcement ratio had a more pronounced influence. Strain analysis of the pure bending region confirmed an approximately linear distribution along the section depth, validating the applicability of the plane section assumption.
(5) From a practical engineering perspective, the results indicate that appropriate incorporation of hybrid fibers can effectively enhance crack resistance and deformation control of BFRP-reinforced lightweight aggregate concrete beams, while reinforcement ratio plays a dominant role in controlling ultimate load capacity. However, the present study is limited to a relatively small number of specimens under short-term loading conditions without statistical replication. Therefore, the results should be interpreted as indicative trends rather than universally applicable design values.

Overall, the experimental findings provide useful insights into the flexural behavior and failure mechanisms of BFRP-reinforced hybrid fiber lightweight aggregate concrete beams, which may serve as a reference for the design and application of lightweight and durable composite structural members.

## Figures and Tables

**Table 1:** Basic physical properties of expanded shale lightweight aggregate.

Particle Size (mm)	Bulk Density (kg/m^3^)	Apparent Density (kg/m^3^)	Porosity (%)	Water Absorption (%)	Cylinder Compressive Strength (Mpa)
5–20	750	1342	41.24	2.9	6.3

**Table 2:** Mechanical properties of BFRP reinforcement.

Diameter (mm)	Tensile Strength (MPa)	Modulus of Elasticity (GPa)
8	921	44.3
12	980	41.9
14	1030	41
16	1069	40.5

**Table 3:** Mix proportions of the reference concrete without fibers.

Cement	Water	Fly Ash	River Sand	Lightweight Aggregate	Superplasticizer
400	160	100	680	532	6

**Table 4:** Basic parameters of test beam.

Specimen Number	Erection Bar	Stirrup	Longitudinal	Reinforcement	HF Content
LAC0-B12	2Φ8	Φ8@100	2B12	1.02%	0%
LAC6-B12			2B12	1.02%	0.6%
LAC9-B12			2B12	1.02%	0.9%
LAC12-B12			2B12	1.02%	1.2%
LAC15-B12			2B12	1.02%	1.5%
LAC12-B8			2B8	0.68%	1.2%
LAC12-B14			2B14	1.56%	1.2%
LAC12-B16			2B16	1.81%	1.2%

**Table 5:** Failure modes of BFRP-reinforced LAC beams under different reinforcement ratios and hybrid fiber contents.

Specimen Number	HF Content	Reinforcement	Failure Mode
LAC0-B12	0%	1.02%	CBF
LAC6-B12	0.6%	1.02%	CDF
LAC9-B12	0.9%	1.02%	CDF
LAC12-B12	1.2%	1.02%	CDF
LAC15-B12	1.5%	1.02%	CDF
LAC12-B8	1.2%	0.68%	BTF
LAC12-B14	1.2%	1.56%	CBF
LAC12-B16	1.2%	1.81%	CBF

**Table 6:** Comparison of experimental and theoretical flexural capacities.

Specimen Number	Calculated Flexural Capacity (kN·m)	Experimental Flexural Capacity (kN·m)	Experimental-to-Calculated Ratio
LAC0-B12	94.31	91.26	0.97
LAC6-B12	96.27	98.52	1.02
LAC9-B12	98.77	108.36	1.11
LAC12-B12	102.53	116.69	1.14
LAC15-B12	105.29	114.51	1.09
LAC12-B8	66.44	68.14	1.03
LAC12-B14	116.57	122.48	1.05
LAC12-B16	134.46	135.37	1.01

## Data Availability

The original contributions presented in this study are included in the article. Further inquiries can be directed to the corresponding author.
